# Bi-directional genetic modulation of GSK-3β exacerbates hippocampal neuropathology in experimental status epilepticus

**DOI:** 10.1038/s41419-018-0963-5

**Published:** 2018-09-20

**Authors:** Tobias Engel, Raquel Gómez-Sintes, Mariana Alves, Eva M. Jimenez-Mateos, Marta Fernández-Nogales, Amaya Sanz-Rodriguez, James Morgan, Edward Beamer, Alberto Rodríguez-Matellán, Mark Dunleavy, Takanori Sano, Jesus Avila, Miguel Medina, Felix Hernandez, José J. Lucas, David C. Henshall

**Affiliations:** 1 Department of Physiology and Medical Physics, Royal College of Surgeons in Ireland, Dublin 2, Ireland; 2 Department of Molecular Neuropathology, Centro de Biología Molecular “Severo Ochoa“ (CBMSO), Consejo Superior de Investigaciones Científicas (CSIC)/Universidad Autónoma de Madrid (UAM) and Centro Investigación Biomédica en Red Enfermedades Neurodegenerativas (CIBERNED), Madrid, Spain; 3 Department of Cellular and Molecular Biology, Centro de Investigaciones Biológicas, CIB-CSIC, C/Ramiro de Maeztu 9, 28040 Madrid, Spain; 4 CIEN Foundation-Queen Sofia Foundation Alzheimer Center and CIBERNED, Instituto de Salud Carlos III Madrid, Madrid, Spain; 5 FutureNeuro Research Centre, Dublin 2, Ireland

## Abstract

Glycogen synthase kinase-3 (GSK-3) is ubiquitously expressed throughout the brain and involved in vital molecular pathways such as cell survival and synaptic reorganization and has emerged as a potential drug target for brain diseases. A causal role for GSK-3, in particular the brain-enriched GSK-3β isoform, has been demonstrated in neurodegenerative diseases such as Alzheimer’s and Huntington’s, and in psychiatric diseases. Recent studies have also linked GSK-3 dysregulation to neuropathological outcomes in epilepsy. To date, however, there has been no genetic evidence for the involvement of GSK-3 in seizure-induced pathology. Status epilepticus (prolonged, damaging seizure) was induced via a microinjection of kainic acid into the amygdala of mice. Studies were conducted using two transgenic mouse lines: a neuron-specific GSK-3β overexpression and a neuron-specific dominant-negative GSK-3β (GSK-3β-DN) expression in order to determine the effects of increased or decreased GSK-3β activity, respectively, on seizures and attendant pathological changes in the hippocampus. GSK-3 inhibitors were also employed to support the genetic approach. Status epilepticus resulted in a spatiotemporal regulation of GSK-3 expression and activity in the hippocampus, with decreased GSK-3 activity evident in non-damaged hippocampal areas. Consistent with this, overexpression of GSK-3β exacerbated status epilepticus-induced neurodegeneration in mice. Surprisingly, decreasing GSK-3 activity, either via overexpression of GSK-3β-DN or through the use of specific GSK-3 inhibitors, also exacerbated hippocampal damage and increased seizure severity during status epilepticus. In conclusion, our results demonstrate that the brain has limited tolerance for modulation of GSK-3 activity in the setting of epileptic brain injury. These findings caution against targeting GSK-3 as a treatment strategy for epilepsy or other neurologic disorders where neuronal hyperexcitability is an underlying pathomechanism.

## Introduction

Epilepsy is one of the most common chronic neurological brain disorders^1^. Despite the development of several new anti-epileptic drugs (AEDs), approximately 30% of patients remain drug refractory^1^. Temporal lobe epilepsy (TLE) is the most common form of epilepsy in adults and is particularly prone to pharmacoresistance and is associated with pathological changes in the hippocampus including neurodegeneration^2^. Status epilepticus (SE) is a prolonged seizure and clinical emergency associated with a high mortality rate and wide-spread brain damage^3^. Similarly to epilepsy, pharmacoresistance in SE remains a serious clinical challenge with ~30% of patients not responding to currently available drugs^1,3^. There is therefore an urgent need to identify new drug targets, preferably with novel mechanisms of action.

Glycogen synthase kinase-3 (GSK-3) is a highly conserved serine/threonine-directed protein kinase^4^. GSK-3 refers to two paralogs, GSK-3α and GSK-3β, which share a highly conserved catalytic domain, but differ at both termini and are encoded by separate genes, with GSK-3β particularly highly expressed in the brain^5^. GSK-3 is present in all brain cell types, where it is highly expressed in the cytoplasm. GSK-3 is, however, present in other cellular compartments including the nucleus, mitochondria and synapses^6–8^. The regulation of GSK-3 is complex and includes autophosphorylation, substrate priming (pre-phosphorylation), association to different protein complexes, and subcellular localization. Inhibitory serine phosphorylation (Ser21 for GSK-3α and Ser9 for GSK-3β) is the most frequently suggested mechanism regulating GSK-3 activity^8^. GSK-3, in particular GSK-3β, has more predicted substrates than any other kinase (>100)^9^. Consequently, GSK-3β has been implicated in the regulation of numerous cellular processes including cellular survival, synaptic reorganization, inflammation, and long-term potentiation (LTP)^8,10–12^.

GSK-3 has emerged as a potential drug target for an array of diseases ranging from cancer to diabetes, cardiovascular conditions, and neurological disorders^13–17^. Among brain diseases, GSK-3 has been particularly linked to Alzheimer’s disease where it promotes hyperphosphorylation of the microtubule-associated protein Tau^18^. Roles have also been suggested for GSK-3 in Huntington’s disease^19,20^ and psychiatric disorders, including bipolar disorder^21^. Lithium, which has been used to treat bipolar disorder for over 60 years, is a competitive GSK-3 inhibitor^22,23^.

Emerging evidence suggests GSK-3 may influence brain excitability and seizure-induced pathology^24–29^. Pathways which directly regulate GSK-3 activity, such as the pro-survival Akt/mammalian target of rapamycin (mTOR) or Wingless-type (Wnt)/β-catenin signalling pathway are strongly associated with epilepsy^30,31^. Early studies showed a protective effect of GSK-3β inhibition against glutamate-induced toxicity in vitro and in vivo^32^ and GSK-3β is de-phosphorylated by the protein phosphatase laforin which is mutated in the progressive myoclonus epilepsy syndrome Lafora disease^24^. Studies of GSK-3β activity indicate that seizures may promote inhibition via Ser9 phosphorylation^8,10,12,26,33^. Conversely, seizures have been reported to result in calpain-mediated truncation of GSK-3, which is predicted to increase GSK-3 activation^34,35^. GSK-3 has also been linked to mossy fiber sprouting^25,26^. Functional studies have resulted in mixed findings. The GSK-3 inhibitor thiadiazolidindione (TDZD-8) protects against seizure-induced damage^27,28^. Valproic acid, a commonly used AED, has been reported to inhibit GSK-3^36^. In contrast, lithium is long-established as having proconvulsant effects when combined with the cholinergic agonist pilocarpine in models of SE^37^. In humans, lithium has been reported to either act as a proconvulsant^38,39^ or anticonvulsant^40^.

There have been no genetic studies carried out to assess the contribution of GSK-3β to seizures and seizure-induced neuropathology relevant to epilepsy. The present study shows that both increased and decreased GSK-3β activity exacerbates seizure-induced cell death, indicating a narrow tolerance for manipulation of this pathway in epilepsy.

## Materials and methods

All reagents and antibodies were purchased from Sigma-Aldrich, Dublin, Ireland, if not stated otherwise.

### Transgenic animal models

All animal procedures were performed in accordance with the principals of the European Communities Council Directive (86/609/EEC) and National Institute of Health’s Guide for the Care and Use of Laboratory Animals. All studies involving animals were approved by the Research Ethics Committee of the Royal College of Surgeons in Ireland (REC 205 and 1322) and the Centro de Biología Molecular Severo Ochoa Institutional Animal Care and Utilization Committee (Comité de Ética de Experimentación Animal del CBM, CEEA-CBM), Madrid, Spain (PROEX293/15). Animal housing and maintenance protocols followed the guidelines of the Council of the European Convention ETS123 and were performed in accordance with the principals of the European Union adopted Directive (2010/63/EU). All transgenic mouse lines are bred on a C57BL/6J background. To establish the role of GSK-3β during SE two different genetic strategies were employed: mice overexpressing GSK-3β (GSK-3β)^41^ and mice expressing a dominate-negative version of GSK-3β (GSK-3β-DN)^42^. GSK-3β and GSK-3β-DN mice were generated as previously described^41,43^. Briefly, GSK-3β mice result from the breeding of TetO mice (bidirectional tet-responsive promoter followed by GSK-3β and β-galactosidase (β-Gal) complementary DNAs, one in each direction) with CamKIIα-tTA (tetracycline-regulated transactivator) mice. The double transgenic mice are designated GSK-3β and overexpress GSK-3β in cortical and hippocampal neurons. The model is based on an inducible promoter that allows postnatal upregulation of GSK-3β^44^. Neuronal transgene expression is achieved via the tTA, which is under the control of the calcium/calmodulin kinase IIα promoter and which binds to the tet-responsive promoter (tetO) driving the expression of both GSK-3β and the reporter gene β-Gal. Hippocampal overexpression of GSK-3β leads to an approximately 25% increase in GSK-3 activity in the hippocampus^45^. GSK-3β mice show impairment in spatial memory^45,46^; GSK-3β overexpression, however, has no effect on body weight (Supplementary Figure 1a) or mortality in the first 12 months of life (data not shown).

To suppress GSK-3β activity in GSK-3β-DN mice, a mutated version of GSK-3β carrying the K85R mutation^47^ is overexpressed in forebrain neurons using the same strategy used for GSK-3β-overexpressing mice^43^. This leads to an approximately 10% reduction in GSK-3 activity in the hippocampus of GSK-3β-DN mice^43^. Mice expressing DN-GSK-3β also show impaired motor coordination on the Rotarod. No difference, however, can be observed on general motor activity using the open field^43^. Similar to GSK-3β-overexpressing mice, no change in body weight (Supplementary data 1B) or life expectancy can be observed in GSK-3β-DN mice when compared to wild-type mice (data not shown).

Fas-deficient *Lpr* mice were obtained from Jackson Laboratories (B6.MRL-Faslpr/J, stock number: 000482).

### Animal model of SE

SE was induced in adult male mice (C57Bl/6 wild-type, GSK-3β, GSK-3β-DN, and *Lpr* mice) by a unilateral stereotaxic microinjection of kainic acid (KA) into the amygdala, as described^48^. Briefly, deeply anesthetized mice (isoflurane 3–5% induction and 1–2% maintenance) were affixed with skull-mounted electrodes (Bilaney Consultants Ltd., Sevenoaks, UK) to record surface electroencephalogram (EEG) using a Grass Comet digital EEG (Medivent Ltd., Lucan, Ireland) and Xltek EEG system (Optima Medical Ltd., Guildford, UK). A guide cannula was affixed over the dura (coordinates from Bregma: AP = −0.94; L = −2.85 mm) and the entire skull assembly fixed in place with dental cement. EEG recordings were commenced once mice fully recovered from anesthesia. Then a 31-gauge internal cannula was inserted into the lumen of the guide to inject KA into the amygdala (0.3 μg in 0.2 μl vehicle; phosphate-buffered saline (PBS), pH adjusted to 7.4). Non-seizure control mice received 0.2 μl intra-amygdala vehicle. Lorazepam (6 mg/kg, intraperitoneal) was administered 40 min after KA. Mice were euthanized at different time points after anticonvulsant and brains flash-frozen whole in 2-methylbutane at −30 °C for Fluoro-Jade B (FjB) staining, perfused with PBS and paraformaldehyde (PFA) 4% for immunofluorescence or microdissected and frozen for Western blot and quantitative PCR (qPCR) analysis.

### Drug treatment

The GSK-3 inhibitors NP031112 (Tideglusib, NP12)^49^ and NP060103 (NP103)^50^ were injected with a 2 μl infusion of intracerebroventricular (i.c.v.) Dimethyl sulfoxide (DMSO) 30 min before intra-amygdala KA to reach a final concentration of 100 µM and 1 mM in the ventricle (ventricle volume was calculated as 30 µl). In the vehicle group, animals were injected with 2 μl of sterile DMSO. Tideglusib belongs to the TDZD family and progressed to clinical trials for Alzheimer’s disease and progressive supranuclear palsy^49,51,52^. Decreased phosphorylation of the known GSK-3 target Tau using antibodies (AT100 and Tau-1), which specifically recognizes GSK-3-dependent phosphoepitopes^53,54^, confirmed a reduction of GSK-3 activity in the hippocampus following i.c.v. GSK-3 inhibitor delivery (Supplementary Figure 2a, b).

### EEG analysis

EEG recordings were analyzed either manually by counting high-frequency high-amplitude discharge polyspiking or by uploading EEG into the Labchart7 software (ADInstruments) to calculate total seizure power of the EEG signal^48^.

### Histopathology

Neuronal death was assessed using FjB staining^48^. Briefly, brains were sectioned on a Leica cryostat and 12-µm-thick sections collected at the level of the dorsal hippocampus and stored at −80 °C. For FjB staining, sections were defrosted, post-fixed with 4% PFA, rehydrated, and transferred to a 0.006% potassium permanganate solution followed by incubation with 0.001% FjB (Chemicon Europe Ltd., Chandlers Ford, UK) and mounted in DPX. Hippocampal cell counts (CA1, C3, and DG separately) were the average of two adjacent sections with a ×40 lens by an observer blind to treatment.

### Diaminobenzidine staining

Diaminobenzidine staining was carried out as previously reported^55^. Mice were 4% PFA perfused, brains post-fixed and cryoprotected in 30% sucrose solution, and 30 µm sagittal sections were cut on a Leica cryostat. Next, brain sections were pretreated for 1 h with 1% bovine serum albumin, 5% fetal bovine serum, and 0.2% Triton^™^ X-100 followed by an overnight incubation with primary antibody β-Gal (Promega, Madison, WI, USA). Next, brain sections were incubated in avidin–biotin complex using the Elite^®^ VECTASTAIN^®^ kit (Vector Laboratories). Chromogen reactions were performed with diaminobenzidine and 0.003% hydrogen peroxide for 10 min. Sections were coverslipped with Fluorosave^™^.

### Synaptosome preparation

Synaptosomes were prepared as reported previously^56^. Mouse hippocampi were dissected on ice, and tissue samples (two ipsilateral hippocampi per sample) were homogenized in 10 ml of ice-cold homogenizing buffer (0.32 M sucrose, 1 mM EDTA, 1 mg/ml bovine serum albumin, and 5 mM HEPES, pH 7.4) in a glass-Teflon douncer with ∼10 strokes at 4 °C. Next, samples were centrifuged for 10 min at 3000 × *g* at 4 °C, and supernatant containing cytoplasm and synaptosomes recovered. Samples were again centrifuged for 12 min at 14,000 × *g* at 4 °C, and supernatant discarded. Pelleted synaptosomes were resuspended in 550 μl of Krebs–Ringer buffer (140 mM NaCl, 5 mM KCl, 5 mM glucose, 1 mM EDTA, and 10 mM HEPES, pH 7.4). Then, 450 μl of Percoll (45% (v/v)) was added, and the two components were mixed by gently inverting the tube. After a 2 min spin at 14,000 × *g* at 4 °C, enriched synaptosomes were recovered and resuspended in 1 ml of Krebs–Ringer buffer. Samples were again spun for 30 s at 14,000 × *g*, and supernatant discarded. Finally, pellet containing synaptosomes was resuspended in assay buffer (HEPES–Krebs buffer) and stored at −20 °C.

### Hippocampal microdissection

Microdissection of the three hippocampal subfields CA3, CA1, and DG was carried out as described previously^57^. Briefly, following the separation of the cerebellum, the two hemispheres were separated. Then, using a dissecting microscope, the whole hippocampus (ipsilateral and contralateral) was separated from the cortex. This was then followed by a microdissection of the different hippocampal subfields of the ipsilateral hippocampus. Following the identification of the boundaries between CA1, DG, and CA3, the three subfields were separated and immediately put on dry ice and stored at −80 °C.

### Western blotting

Western blotting was performed as described previously^48^. Following quantification of protein concentration, 30 µg of protein samples were boiled in gel-loading buffer and separated by sodium dodecyl sulfate–polyacrylamide gel electrophoresis. Proteins were transferred to nitrocellulose membranes and probed with the following primary antibodies: β-actin, α-tubulin, GSK-3 (BD Transduction laboratories, Oxford, UK), AT100 and Tau-1 (Innogenetics, Ghent, Belgium), GAPDH, GSK-3β, and P9Ser-GSK-3β (Cell Signaling, Leiden, Netherlands), and synaptophysin (Abcam, Cambridge, UK). Next, membranes were incubated with horseradish peroxidase-conjugated secondary antibodies (Isis Ltd., Bray, Ireland) and protein bands visualized using chemiluminescence (Pierce Biotechnology, Rockford, IL, USA). Gel bands were captured using a Fujifilm LAS-3000 (Fujifilm, Tokyo, Japan) and analyzed using Alpha-EaseFC4.0 software.

### RNA extraction and real-time quantitative polymerase chain reaction

RNA extraction was undertaken as previously described using TRIzol^®^ (QIAzol Lysis Reagent, Qiagen, Hilden, Germany)^48^. One microgram of total RNA was used to generate complementary DNA by reverse transcription using SuperScript^®^ II reverse transcriptase enzyme (Thermo-Fisher, MA, USA). Quantitative real-time PCR was performed using a LightCycler 1.5 (Roche Diagnostics GmbH, Mannheim, Germany) in combination with QuantiTect^®^ SYBR^®^ Green PCR Kit (Qiagen, Hilden, Germany) as per the manufacturer’s protocol, and 1.25 µM of primer pair was used. Data were analyzed by LightCycler 1.5 software, data were normalized to expression of β-actin and represented as relative quantification values. Primers were designed using Primer3 software (http://frodo.wi.mit.edu). Primer sequences: *gsk-3β* (F: tggcgtgtgatgtcaggtat; R: taagctggcatcctgcaacac); *p21*^*WAF/Cip*^ (F: tcccgactcttgacattgct; R: tgcagaaggggaagtatggg); *c-Myc* (F: tcagacacggaggaaaacga; R: cgtctgcttgaatggacagg); *wnt9b* (F: agcttcctctctcaacaccc; R: tttgttggctttctcctcgc); *mcl-1* (F: gaaggcggcatcagaaatgt; R: gcagcttcaagtccaccttc); and *β*-actin (F: gggtgtgatggtgggaatgg; R: ggttggccttagggttcagg).

### Microarray analysis

Microarray studies were undertaken at an Affymetrix authorized service provider (University College Dublin, Dublin, Ireland) as described previously^48^. Total RNA was extracted from wild-type and GSK-3β-overexpressing mice 6 h following SE and was hybridized to the Mouse Genome 430 2.0 Genechip array. Affymetrix GeneChip image files were analyzed by robust multichip analysis using RMAExpress 0.5 (http://rmaexpress.bmbolstad.com). Data were log transformed, and the threshold for significant regulation was set at 1.5-fold to retain genes that exhibit a biologically meaningful level of regulation, but not exclude certain genes that, because of high constitutive expression, may show lower degrees of change. Gene ontology and function were assigned using the two bioinformatic programs DAVID Bioinformatics Resources 6.8 (http://david.abcc.ncifcrf.gov/) and Enrichr^58^.

### Statistical analysis

Data are presented as the mean ± s.e.m. Two group comparisons were made using unpaired Student’s two-tailed *t* test, while multi-group comparisons were made using two-way analysis of variance (ANOVA) followed by post hoc testing using Fisher’s exact test (StatView). Significance was accepted at *p* < 0.05.

## Results

### Spatiotemporal changes in GSK-3β expression and phosphorylation following SE

**Fig. 1:**
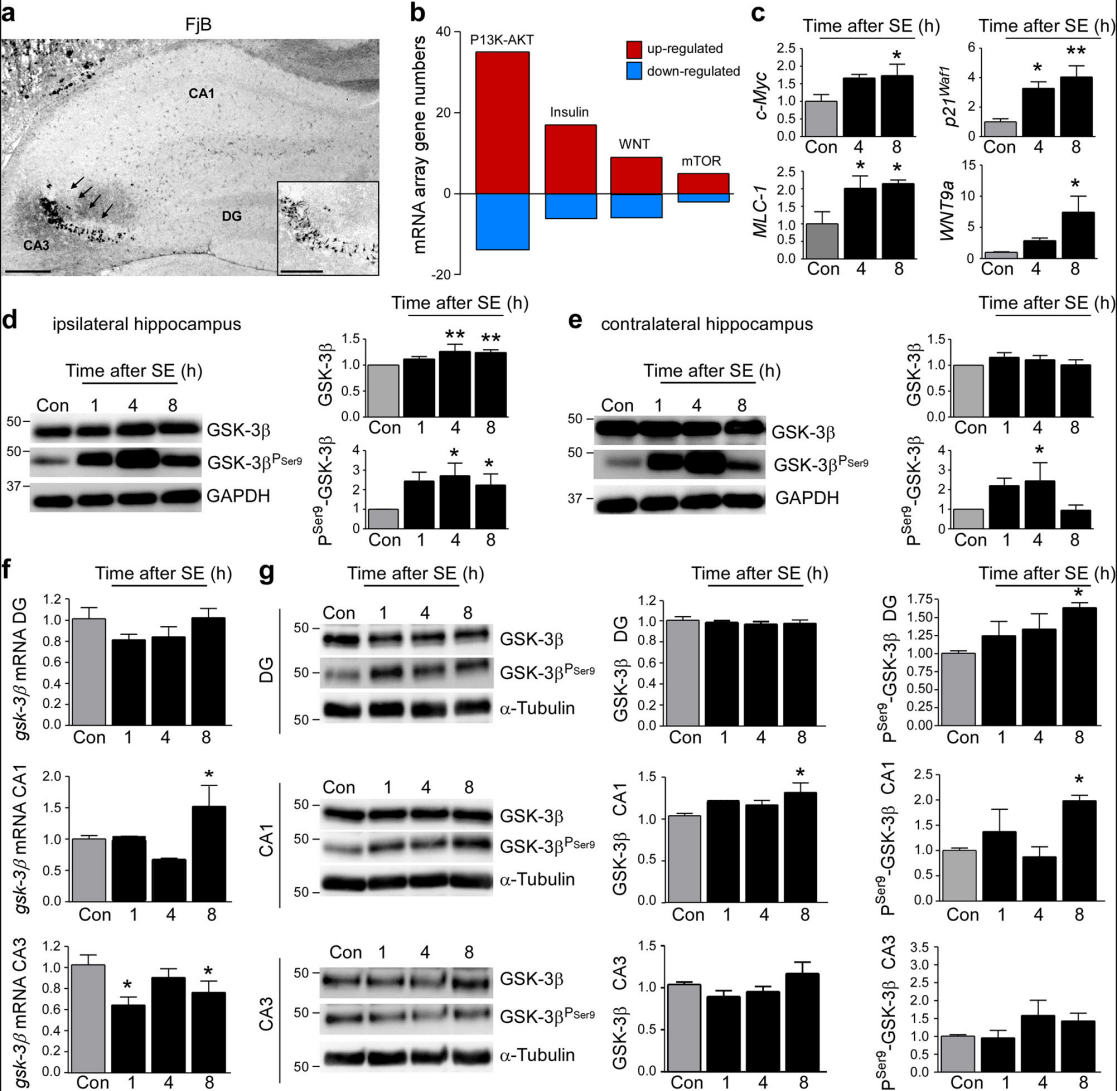
Spatiotemporal changes in hippocampal GSK-3β expression and inhibitory Ser9 phosphorylation following SE. **a** Representative photomicrograph (×5 lens) showing characteristic cell death in the CA3 subfield of the hippocampus (arrows and insert) 24 h following intra-amygdala KA injection in mice. Only sporadic cell death can be observed in the remaining hippocampal subfields (CA1 and DG). Scale bar = 500 µm for overview and 250 µm for insert. **b** Graph showing numbers of genes with altered expression 6 h post SE determined by mRNA array involved in pathways associated with GSK-3 signalling. **c** Graphs showing increased expression of genes encoding for the proteins c-Myc, MCL-1, P21^Waf1^, and Wnt9a in the ipsilateral hippocampus following SE (mean ± s.d., *p < 0.05 and **p < 0.001 by two-way ANOVA with Fisher’s post hoc test; *n* = 4 per group). **d** Representative Western blots (*n* = 1 per lane) and corresponding graphs showing increased expression and Ser9 phosphorylation of GSK-3β in the ipsilateral hippocampus post-SE (mean ± sd, *p < 0.05 and **p < 0.001 by two-way ANOVA with Fisher’s post hoc test; *n* = 4 per group). **e** Western blots (*n* = 1 per lane) showing no apparent changes in GSK-3β expression, however, increased GSK-3β Ser9 phosphorylation in the contralateral hippocampus following SE (mean ± s.d., *p < 0.05 by two-way ANOVA with Fisher’s post hoc test; *n* = 6 per group). **f** Graphs showing *gsk-3β* mRNA levels in the hippocampal subfields DG, CA1, and CA3 following SE (mean ± s.d., *p < 0.05 by two-way ANOVA with Fisher’s post hoc test; *n* = 4 per group). **g** Representative Western blots (*n* = 1 per lane) and corresponding graphs showing SE-induced GSK-3β expression and Ser9 phosphorylation changes in the hippocampal subfields DG, CA1. and CA3 (mean ± s.d., *p < 0.05 by two-way ANOVA with Fisher’s post hoc test; *n* = 4 per group). *p < 0.05. DG dentate gyrus, CA cornu ammonis

To explore the response of GSK-3β to prolonged seizures, we used a well-characterized model of intra-amygdala KA-induced SE in mice^59^. As previously reported, SE resulted in hippocampal damage that was mainly localized to the ipsilateral CA3 subfield, although scattered cell death was present in the CA1 and the hilus regions (Fig. 1a)^59^. Neuronal death was not observed in the contralateral hippocampus or in vehicle-injected mice, as described^59^.

To explore whether seizures in this model triggered changes in signalling pathways regulated by GSK-3, we interrogated a previously published gene array profile of the model^48^. We focused our analysis on the PI3K/Akt, Wnt, insulin, and the mTOR pathways that are linked to both epilepsy and GSK-3 function^8,60–63^. Interrogation of the data identified changes in the expression of multiple genes associated with each pathway. The P13K/Akt pathway showed the largest number of genes undergoing SE-induced changes (Fig. 1b and Supplementary information Table 1). An increase in gene expression was the predominant response among all four selected pathways (Fig. 1b). These results were validated using individual qPCR for a subset of genes previously associated with GSK-3, including the myelocytomatosis oncogene (c-Myc), myeloid cell leukemia sequence-1 (MCL-1), cyclin-dependent kinase inhibitor 1 (p21^Waf1^), and the Wnt signalling pathway member Wnt9a^9^ (Fig. 1c). Together, these findings demonstrate that SE modulates expression of numerous pathways linked to GSK-3.

We next investigated whether SE directly affects GSK-3β expression and activity. SE led to an increase in GSK-3β protein levels in the ipsilateral hippocampus (Fig. 1d). We also detected a strong increase in GSK-3β Ser9 phosphorylation following SE, consistent with inhibition of GSK-3β activity (Fig. 1d). Ser9 GSK-3β phosphorylation was also increased in the contralateral hippocampus, although GSK-3β expression levels were unchanged (Fig. 1e).

We next separately analyzed each ipsilateral hippocampal subfield. GSK-3β transcription was increased in CA1, decreased in CA3, and no changes were observed in the DG (Fig. 1f). In line with an increase in GSK-3β transcription, increased GSK-3β expression was also found in the ipsilateral CA1 subfield (Fig. 1g). No significant changes in GSK-3β expression were observed for the other subfields (Fig. 1g). Thus, the upregulation of GSK-3β following SE appears to be driven by changes within the CA1 subfield. Increased phosphorylation at Ser9 of GSK-3β was restricted to the CA1 and DG hippocampal subfields. GSK-3β phosphorylation was not changed in the damage-vulnerable ipsilateral CA3 subfield (Fig. 1g). Together, our results establish a subfield-specific spectrum of transcriptional and post-transcriptional responses of GSK-3β in the hippocampus following SE.

### GSK-3β overexpression exacerbates seizure-induced cell death

**Fig. 2:**
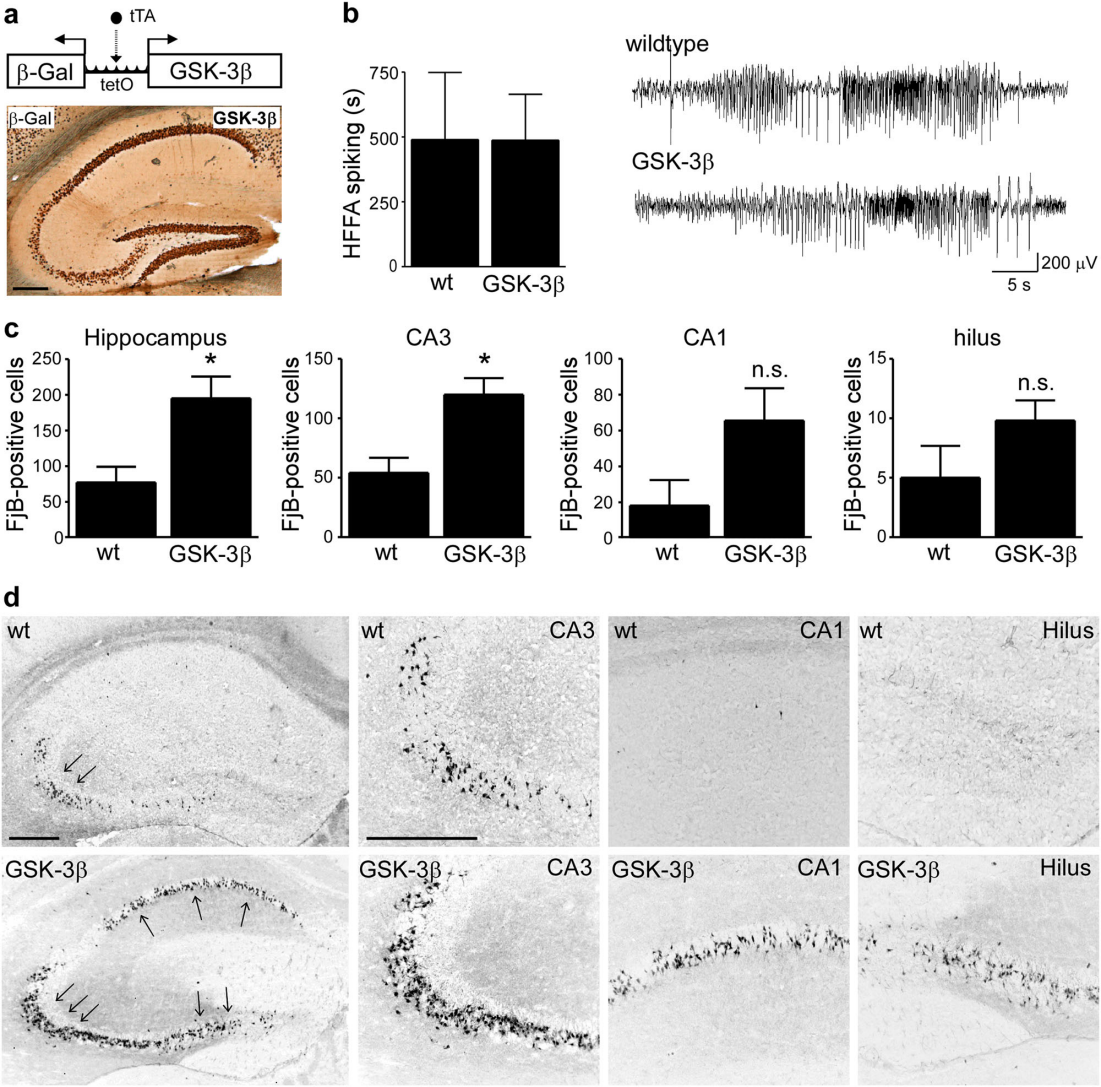
Increased neurodegeneration in GSK-3β-overexpressing mice following SE. **a** Schematic showing strategy to overexpress GSK-3β in neurons and photomicrograph (×5 lens) showing reporter gene expression (β-Gal) in the hippocampus. Scale bar = 500 µm. **b** No significant differences in high-frequency high-amplitude (HFHA) spiking between GSK-3β-overexpressing mice and wild-type (wt) mice during SE (mean ± s.d., *p* = 0.99, by Student’s two-tailed *t* test, *n* = 3 (wt) and 5 (GSK-3β)). **c** Graph showing increased neurodegeneration in the hippocampus in mice overexpressing GSK-3β when compared to wild-type mice 72 h following SE with the CA3 subfield of the hippocampus of GSK-3β-overexpressing mice showing the highest increase in seizure-induced cell death (mean ± s.d., *p < 0.05 by Student’s two-tailed *t* test, *n* = 5 (wt) and 11 (GSK-3β). **d** Representative photomicrographs (×5 and ×20 lens) of FjB staining in the hippocampus of wt and GSK-3β-overexpressing mice 72 h following SE. Scale bar = 500 µm for hippocampal overview and for hippocampal subfields. n.s. not significant

Since GSK-3β inhibition was a feature of hippocampal subfields spared from seizure-induced cell death, we hypothesized that an increase in GSK-3β activity would increase seizure-induced neuronal death in this model. To test this, we used a transgenic mouse model which specifically overexpresses GSK-3β in forebrain neurons (Fig. 2a)^41^.

We first explored whether increased GSK-3β expression has effects on the duration or severity of SE. Cortical EEG analysis covered the 40 min from intra-amygdala KA injection until the administration of anticonvulsant. The duration of HAHFDs, which are associated with seizure-induced cell death in the model, was not different between wild-type and GSK-3β-overexpressing mice (Fig. 2b). These findings indicate that overexpression of GSK-3β is not sufficient to change general seizure susceptibility in this model.

Next, the hippocampus from these mice was examined using the neuronal cell death marker FjB. Wild-type mice showed the typical lesion in the CA3 subfield with only scattered cell death in the remaining hippocampal subfields (Fig. 2c, d). In contrast, mice overexpressing GSK-3β displayed significantly increased seizure-induced neuronal death in the hippocampus, particularly within the CA3 subfield (Fig. 2c, d). Thus, consistent with our hypothesis, GSK-3β overexpression in neurons increases seizure-induced neurodegeneration.

### GSK-3β overexpression during SE impacts on the expression of genes involved in inflammatory signalling and synaptic transmission

To explore possible mechanisms by which GSK-3β overexpression promotes neurodegeneration during SE, we performed genome-wide analysis of gene expression in the hippocampus of wild-type and GSK-3β-overexpressing mice subjected to SE.

**Fig. 3:**
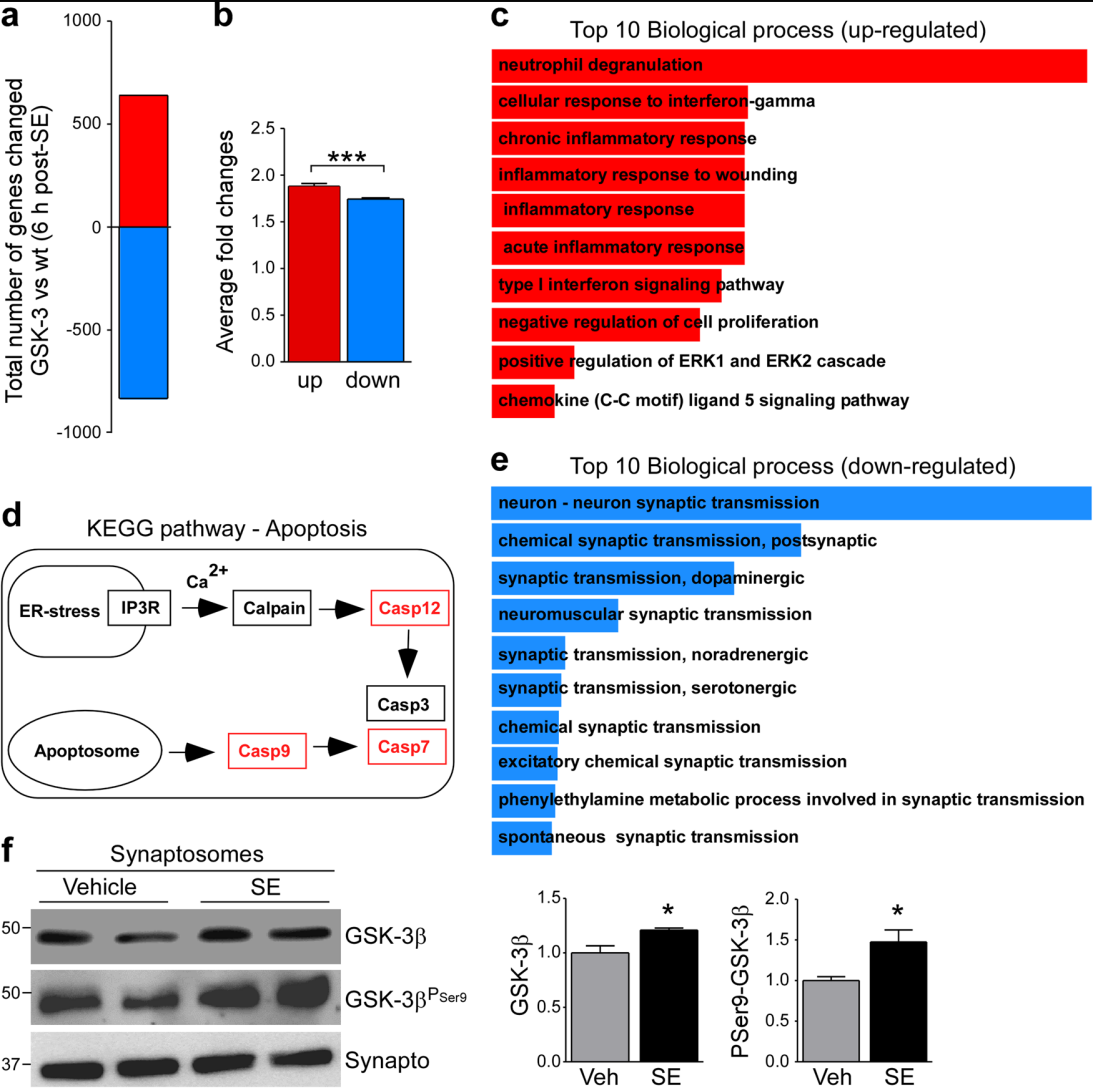
Increased expression of apoptosis-inducing genes in mice overexpressing GSK-3β following SE. **a** Microarray analysis found more genes down-regulated in GSK-3β mice when compared to wild-type mice than upregulated following SE. **b** Graph showing higher fold-change in upregulated gene pool when compared to down-regulated gene pool of genes showing altered expression in GSK-3β mice when compared to wild-type mice after SE (mean ± s.d., ***p < 0.001 by Student's two tailed *t* test, *n* = 642 (up) and 832 (down)). **c** Diagram showing top ten biological processes of the upregulated gene pool in GSK-3β mice subjected to SE determined by bioinformatic program Enrichr. **d** KEGG pathway showing caspases (Casp, red) with higher fold increase in GSK-3β mice. ER endoplasmic reticulum, IP3R inositol trisphosphate receptor. **e** Diagram showing top ten biological processes of the down-regulated gene pool in GSK-3β mice subjected to SE determined by bioinformatic program Enrichr. **f** Western blots (*n* = 2 per lane) and graphs showing increased GSK-3β expression and GSK-3β Ser9 phosphorylation in synaptosomes isolated from the ipsilateral hippocampus 8 h following SE (mean ± s.d., *p < 0.05 by Student’s two-tailed *t* test, *n* = 4)

From the total of genes called present in the mouse hippocampus, 1474 displayed at least a 1.5-fold-change in expression between wild-type and GSK-3β-overexpressing mice (Supplementary information data set 1). Overall, GSK-3β overexpression resulted in down-regulation of more genes than upregulation (Fig. 3a). However, the average fold-change was higher in genes with an increased expression (Fig. 3b). To establish which pathways and target genes were altered by GSK-3β overexpression following SE, we used two bioinformatics tools, Enrichr^58^ for pathway analysis and the DAVID Bioinformatics Resources to identify specific target genes. Using Enrichr and analyzing two sets of gene pools consisting of genes which were found to be upregulated and genes which were found to be down-regulated in GSK-3β-overexpressing mice subjected to SE, we found that in mice overexpressing GSK-3β, transcripts involved in inflammatory processes were particular abundant, in line with GSK-3β driving inflammation^11^ (Fig. 3c and Supplementary information Table 2). Interestingly, using DAVID Bioinformatics Resources we found that there was a strong signal associated with control of apoptosis, including the upregulation of caspase-7, -9, and -12 in GSK-3β-overexpressing mice (Fig. 3d). Despite the lack of effects of GSK-3β overexpression on electrographic seizures, we identified a number of down-regulated genes involved in synaptic transmission in GSK-3β-overexpressing mice. This suggests that GSK-3β may directly affect excitability, consistent with known inhibitory effects of increased GSK-3 on LTP^10,12^ (Fig. 3e and Supplementary information Table 2). In support of a link to synaptic function, we observed that SE led to an enrichment of GSK-3β protein in the synaptosomal compartment (Fig. 3f). Interestingly, synaptosomal Ser9 phosphorylation of GSK-3β was highly increased following SE (Fig. 3f).

In summary, GSK-3β overexpression during SE promotes an increase in transcription of genes involved in inflammatory processes and a down-regulation in genes involved in synaptic transmission.

### Pharmacological inhibition of GSK-3 exacerbates seizure-induced cell death during SE

To test a potential neuroprotective effect of GSK-3 inhibition during SE, mice were treated with two highly specific, structurally different GSK-3 inhibitors before the injection of intra-amygdala KA (Tideglusib (NP031112, NP12) and NP060103 (NP103)).

**Fig. 4:**
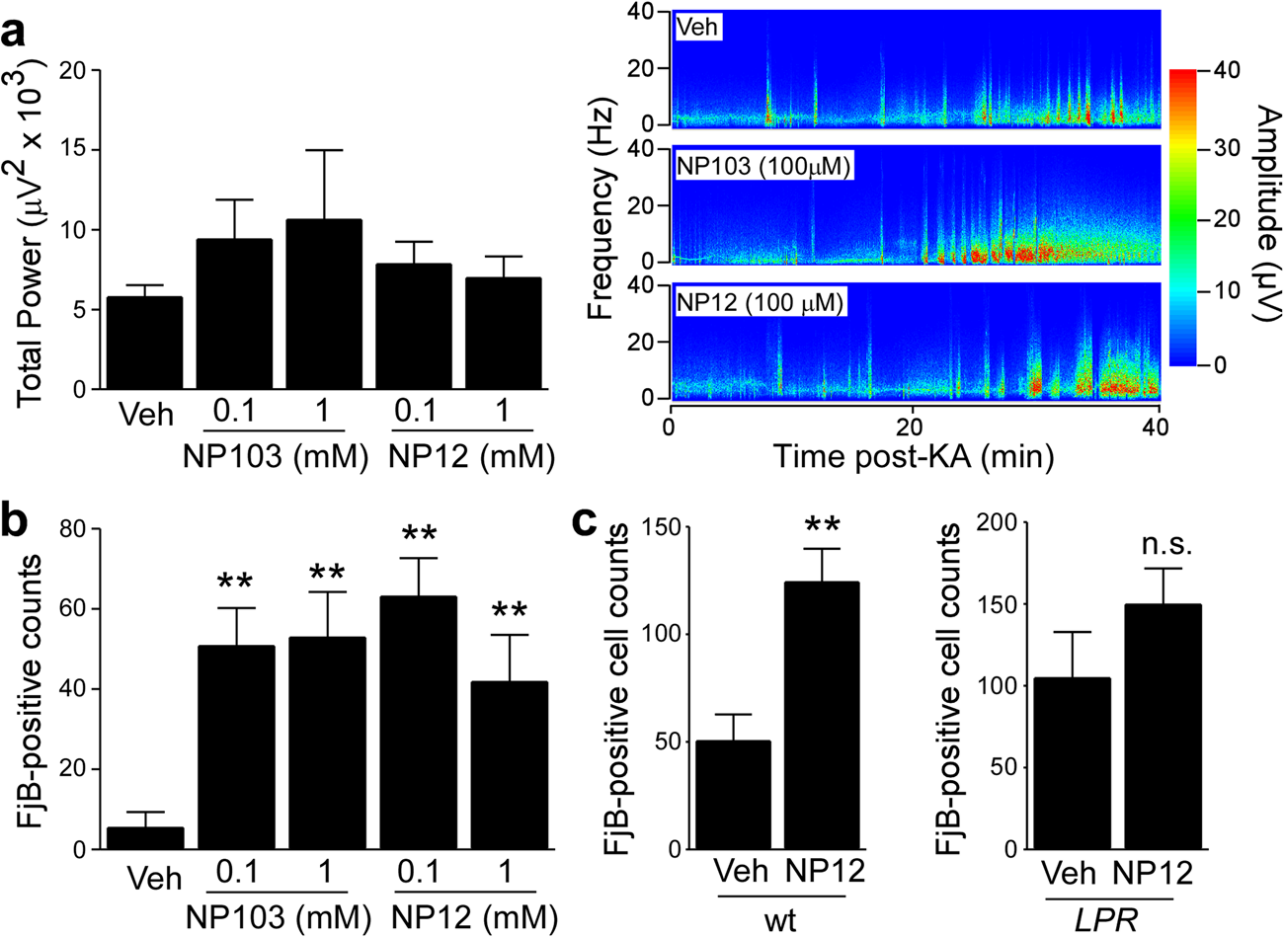
Pharmacological GSK-3 inhibition exacerbates seizure-induced cell death. **a** Graph showing similar seizure total power during a 40 min recording period starting at the time of intra-amygdala KA injection until administration of lorazepam between mice treated with vehicle or with the GSK-3 inhibitors NP103 or NP12 (mean ± s.d., *p < 0.05 by two-way ANOVA with Fisher’s post hoc test; *n* = 10 per group). **b** Graph showing increased neurodegeneration in the ipsilateral hippocampus in mice treated with the GSK-3 inhibitors NP103 or NP12 when compared to vehicle-treated mice 24 h following SE (mean ± s.d., *p < 0.05 and **p < 0.01 by two-way ANOVA with Fisher’s post hoc test; *n* = 10 per group). **c** Graphs showing increased ipsilateral hippocampal cell death in *Lpr* wt mice treated with GSK-3 inhibitor NP12 when compared to vehicle *Lpr* wild-type mice 24 h following SE. No significant difference can be observed between Fas knockout mice (*Lpr*) treated with GSK-3 inhibitor NP12 when compared to vehicle-treated Fas knockout mice 24 h following SE (mean ± s.d., **p < 0.01 by Student’s two-tailed *t* test, *n* = 7 (wt Veh), 8 (wt NP12), 7 (*Lpr* Veh), and 9 (*Lpr* NP12)). n.s. not significant

Neither Tideglusib nor NP103 had a significant effect on seizure severity during SE (Fig. 4a). Analysis of the hippocampus of mice given either GSK-3 inhibitor revealed, however, increased neurodegeneration in the hippocampus following SE (Fig. 4b).

We next sought to explore the mechanism by which inhibition of GSK-3 increased seizure-induced neuronal death. GSK-3 has been shown to promote cell death via the intrinsic (mitochondrial) apoptosis pathway as well as protect against apoptosis through blocking the extrinsic apoptosis pathway mediated by tumor necrosis factor (TNF) receptor family members such as Fas^64^. Notably, TNF signalling components are upregulated in brain tissue from TLE patients and inhibiting this pathway is neuroprotective in experimental seizure models^65,66^. In line with GSK-3 inhibition promoting cell death via extrinsic Fas signalling, two recent studies showed that loss of Fas protected against GSK-3 inhibition-induced neuronal death^42,67^. Therefore, to test whether the increased seizure-induced cell death observed in mice treated with GSK-3 inhibitors is mediated via Fas signalling, we studied their effects in mice deficient for Fas treated with NP12 or vehicle. Confirming our previous findings, GSK-3 inhibition led to increased seizure-induced neuronal death in wild-type mice subjected to SE (Fig. 4c). In contrast, hippocampal cell death was similar between GSK-3 inhibitor and vehicle-treated Fas knockout mice (Fig. 4c). Taken together, these results suggest that increased seizure-induced neuronal death in response to GSK-3 inhibition is at least in part mediated by the Fas extrinsic cell death pathway.

### Genetically reduced GSK-3 activity increases seizure pathology during SE

**Fig. 5:**
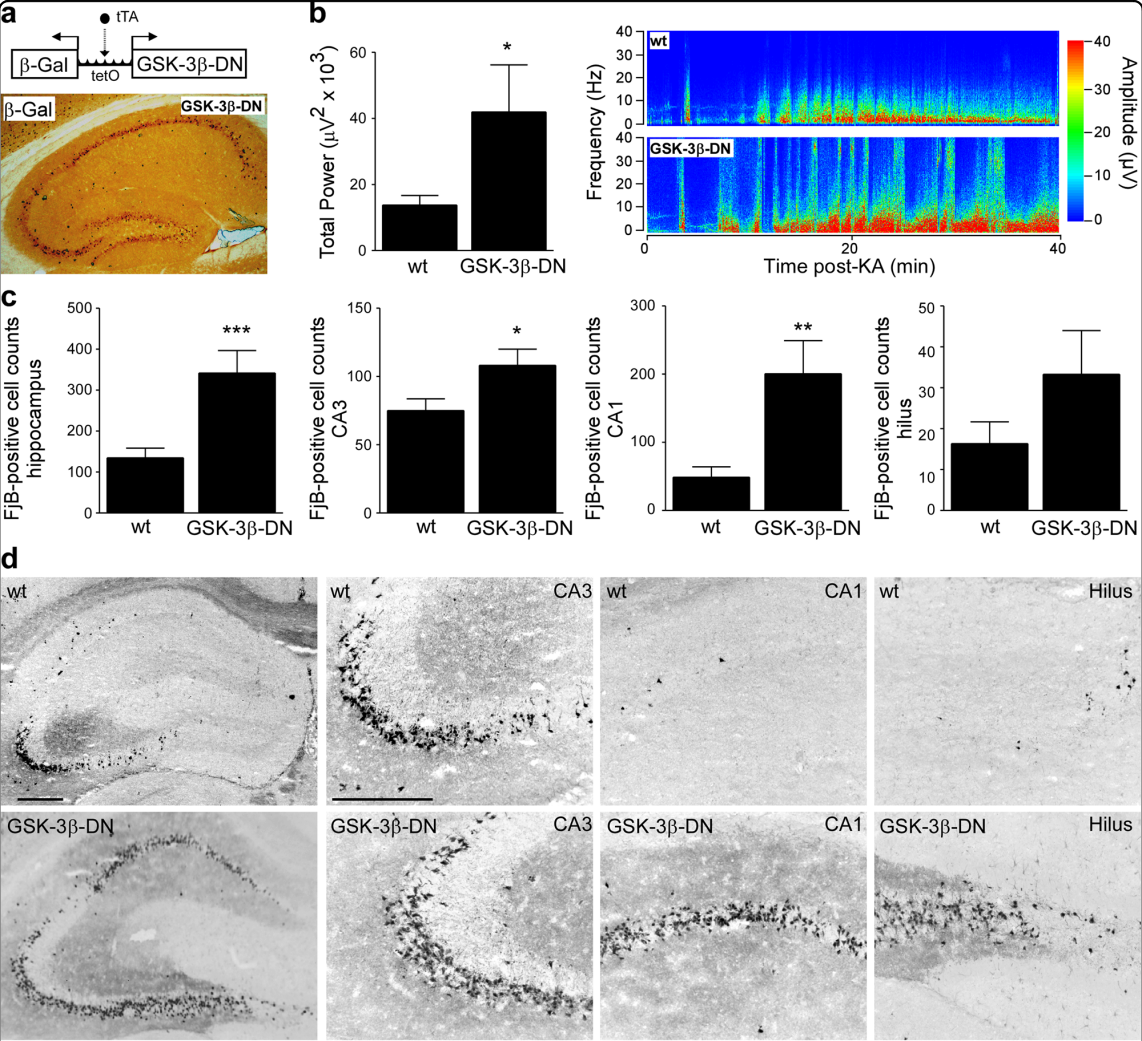
Impact of GSK-3β-DN expression on seizure severity and neurodegeneration. **a** Schematic of transgenic strategy to overexpress GSK-3β-DN in forebrain neurons and representative immunostaining showing the expression of the reporter gene β-Gal in the hippocampus. Scale bar = 500 µm. **b** Graph and representative heatmap showing increased total seizure power of GSK-3β-DN mice when compared to wild-type (wt) mice during a 40 min recording period starting at intra-amygdala KA injection until lorazepam administration (mean ± s.d., *p < 0.05 by Student’s two-tailed *t* test, *n* = 9 (wt) and *n* = 7 (GSK-3β-DN)). **c** Graph and corresponding FjB stainings (×5 and ×20 lens) showing strongly increased neurodegeneration in the hippocampus of GSK-3β-DN mice when compared to wild-type (wt) mice 72 h following SE (mean ± s.d., *p < 0.05, **p < 0.01 and ***p < 0.001 by Student’s two-tailed *t* test, *n* = 12 (wt) and 6 (GSK-3-DN)). Scale bar = 500 µm for hippocampal overview and for hippocampal subfields

To support our pharmacological results, we sought genetic evidence that inhibition of GSK-3 promotes seizure-induced neuronal death in vivo. For this, we used a recently developed mouse model which overexpresses a dominant-negative form of GSK-3β in forebrain neurons (GSK-3β-DN mice), reducing the activity of both GSK-3β and GSK-3α (Fig. 5a)^43^. When subjected to SE induced by intra-amygdala KA, GSK-3β-DN mice showed a >50% increase in seizure severity during SE (Fig. 5b). Analysis of hippocampal sections of GSK-3β-DN mice following SE revealed increased seizure-induced neurodegeneration throughout the hippocampus, which was particularly severe in the CA1 and CA3-hilus subfields (Fig. 5c, d). Therefore, this genetic approach reveals an additional aspect of GSK-3 function not observed with pharmacological inhibitors, that reduction of GSK-3 activity during SE increases seizure severity and the resulting seizure-induced pathology.

## Discussion

The present study provides genetic as well as pharmacological evidence that GSK-3 can influence seizure severity and seizure-induced brain pathology. The main finding was that both an increase as well as a decrease in the activity of GSK-3β exacerbates seizure-induced brain damage. Taken together, the study indicates a narrow tolerance for GSK-3 manipulation and argues against targeting this enzyme for the treatment of SE and attendant epileptic brain injury.

The present study shows that prolonged seizures cause a temporal and subfield-specific expression and activation pattern of GSK-3β in the hippocampus. We also observed a strong increase in Ser9 phosphorylation that is known to inhibit kinase activity. While SE led to an increase in GSK-3β expression in the CA1 subfield of the hippocampus, GSK-3β transcript levels were decreased in CA3. The decrease in CA3 was not, however, accompanied by a decrease in protein levels, possibly due to seizure-induced inhibition of the ubiquitin-proteasome system^59^. Overall, these findings extend earlier reports that seizures increase GSK-3 expression within the hippocampus while also having an inhibitory action on GSK-3β^8,10,12,26^. By using a model in which there is divergent damage within the ipsilateral hippocampus, we could further demonstrate that protected brain regions display increased inhibition after seizures. It is uncertain why GSK-3β expression was particularly increased in the CA1 subfield and what drives this response. The cause of the decrease in CA3 is perhaps via damage-induced impairment of transcription and translation since the CA3 is the main site of pathology in the model^68^. The decrease in GSK-3β transcription in CA3 may also represent an intracellular survival mechanism since GSK-3β overexpression leads to an increase in seizure-induced neurodegeneration. As to what functional consequences an increase in GSK-3β expression in CA1 represents and why this expression increase does not lead to cell death remains elusive. The most likely explanation is the concordant increase in inhibitory phosphorylation status, with Ser9 phosphorylation being increased in CA1 and DG. This would effectively counter the risk of elevated GSK-3-promoting neurodegeneration. While inhibitory GSK-3 phosphorylation is the most studied process controlling GSK-3 activity, it is, however, not the only one. Other post-translational mechanisms have been described including substrate priming, the incorporation of GSK-3 into protein complexes and subcellular localization^8^. GSK-3β has been shown to be truncated by calpains at the N-terminal end removing the inhibitory Ser9 phosphorylation side, thereby increasing GSK-3β activity^34^. Notably, we previously showed in the intra-amygdala KA mouse model that this was most evident in the CA3 subfield^35^, possibly contributing to seizure-induced neurodegeneration. GSK-3β truncation in CA3 may also be the reason why this specific subfield showed no increase in GSK-3β Ser9 phosphorylation.

A key finding of our study was in vivo genetic evidence that GSK-3β overexpression can promote seizure-induced neuronal death. A proapoptotic role of GSK-3β has been well described, with GSK-3β driving the intrinsic apoptotic signalling pathway^64^. Our findings support GSK-3β as an additional mediator of seizure-induced neuronal death linked to the intrinsic pathway, which includes various Bcl-2 family proteins and mitochondrial components^69^. Notably, analysis of gene expression responses to increased GSK-3 during SE showed a strong signal for members of the caspase family^70^. Thus, GSK-3β may also be involved in the promotion of intrinsic apoptotic pathways during SE. Whether GSK-3β directly regulates the transcription of caspases is unknown, although transcription factors are by far the largest protein family targeted by GSK-3^8^. Gene profiling also showed GSK-3β overexpression during SE increased genes involved in inflammatory processes. This is consistent with early links between GSK-3 and inflammatory signalling pathways^71^ and evidence that GSK-3 inhibitors reduce inflammation in various disease models^6^. Indeed, there remains strong interest in GSK-3 as a target to control inflammatory processes^72^. In the brain, GSK-3 has been found to drive the production of pro-inflammatory cytokines such as TNF-α, interleukins (e.g., interleukin-1β (IL-1β), IL-6), interferons, or chemokines released by glia, possibly through the regulation of transcription factors including nuclear factor kappa-light chain enhancer of activated B cells or signal transducer and activator of transcription-3^72^. Neuroinflammatory processes have been repeatedly shown to be activated in both experimental and human epilepsy, including the release of cytokines such as Il-1β and TNF-α. Moreover, inflammation-interfering drugs reduce seizure severity and brain damage in experimental models of epilepsy^73,74^. Our data suggest, therefore, that an increase in GSK-3 activity may contribute to the seizure-induced pro-inflammatory state in the brain exacerbating brain pathology.

The second major finding here was that GSK-3 inhibition during SE also increases damage to the brain. These results were unexpected and have important implications for efforts to develop treatments for brain diseases based on targeting this enzyme. This apparently contradictory finding may be explained by the previously elucidated dual and opposing effects of GSK-3 on apoptosis signalling pathways. GSK-3 has been shown to promote the intrinsic apoptotic pathway but also to inhibit the extrinsic apoptotic pathway^64^. Both are activated by seizures and have been previously linked to seizure-induced cell death^65,66,75,76^.

Recent work showed that GSK-3 inhibition can lead to neurodegeneration in a Fas receptor-dependent manner^42,43,67^. Our investigation of the mechanism by which GSK-3 inhibition promotes seizure-induced neuronal death is consistent with those findings. Specifically, GSK-3 inhibition did not increase cell death in mice lacking the Fas receptor. In contrast to our findings, a previous study using a GSK-3 inhibitor (TDZD-8) belonging to the same drug family as NP12, which was used in our study, provided protection against seizure-induced cell death in a mouse model of intraperitoneal KA-induced SE^28^. In addition, no difference in seizure severity was reported^28^. The reason for these discrepancies is uncertain but may relate to differences in animal models (intra-amygdala KA vs. intraperitoneal KA) or drug delivery route (i.c.v. vs. intraperitoneal), which could result in quite different local drug concentrations within the target tissues. Nevertheless, our use of two different drugs at two doses in combination with a genetic approach provides strong evidence that GSK-3 inhibition leads to an increase in neurodegeneration in the tested model.

Finally, some of our data support GSK-3 having a direct role in the control of brain excitability. Specifically, genetic inhibition of GSK-3 led to increased seizure severity during SE. This finding is consistent with other emerging work linking GSK-3 to synaptic plasticity. For example, increased GSK-3 has been reported to promote long-term depression, whereas GSK-3 inhibition can promote LTP^10,12^. Several mechanisms have been proposed including modulation of γ-aminobutyric acid (GABA)_A_ and *N*-methyl-d-aspartate receptors^10,77^. Notably, our gene expression profiling study shows that GSK-3 overexpression leads to a suppression of genes implicated in synaptic transmission. This suggests that GSK-3β may act as a break on processes leading to an increase in the expression of genes implicated in neurotransmission, and that by removing this break via GSK-3 inhibition, genes facilitating seizure generation are upregulated. We also noted that GSK-3 accumulates within synaptic structures after SE where it may act locally to alter activity of targets. The fact that seizures lead to an increase in synaptic GSK-3β levels further strengthens a possible role of GSK-3 during synaptic transmission during seizures. Interestingly, the strong increase in inhibitory Ser9 phosphorylation of GSK-3 within this compartment following SE further suggests this being proconvulsant, as GSK-3 inhibition increased seizure severity during SE. It is unclear why overexpression of GSK-3β, in contrast to GSK-3 suppression, leads to an increase in seizure-induced cell death without altering seizure severity. There may be a specific activity threshold, not reached in the present study, that must be exceeded for differences in GSK-3 activity to alter both seizures and seizure-induced cell death. Although seizure damage is loosely correlated with seizure duration in the intra-amygdala KA model^78^, other manipulations of cell death-regulatory genes have been shown to modulate either the seizures or the damage but not both^79,80^. Future studies could test whether further elevating GSK-3, perhaps via delivery of the gene via a viral approach, can increase seizures and resolve this apparent discrepancy. Another explanation may be differences in GSK-3 down-stream targets depending on the GSK-3 activity status. Exploring these differences, for example, by using mass spectrometry, and how exactly GSK-3 influences neuronal survival and neuronal transmission during seizures has, however, not been further explored and must be addressed in future studies. While our studies have focused primarily on GSK-3β, GSK-3α may also carry out a functional role during seizures. GSK-3 inhibitors impact on both isoforms and the activity of both GSK-3β and GSK-3α is down-regulated in GSK-3β-DN-expressing mice. GSK-3α-knockout mice are available^81^ and the impact of a specific modulation of GSK-3α should be addressed in future studies.

In summary, our findings demonstrate GSK-3 is important in seizure-generation and seizure-induced pathology. Caution must be exercised when targeting GSK-3 as a possible treatment where brain hyperexcitability is one of the main underlying pathological characteristics of the disease.

## Electronic supplementary material

Supplementary Figure 1 and 2

Supplementary Information Table 1

Supplementary Information Table 2

Supplementary Data set 1
