# Supplementary material for: Bi-directional genetic modulation of GSK-3β exacerbates hippocampal neuropathology in experimental status epilepticus
Source: Cell Death Dis. 2018 Sep 20;9(10):969. doi: 10.1038/s41419-018-0963-5 (PMC6147910; doi:10.1038/s41419-018-0963-5)
Supplement: Supplementary file 1 — Supplementary Figure 1 and 2 [file 41419_2018_963_MOESM1_ESM.docx]

**Supplementary Material**

**Supplementary Figure 1**


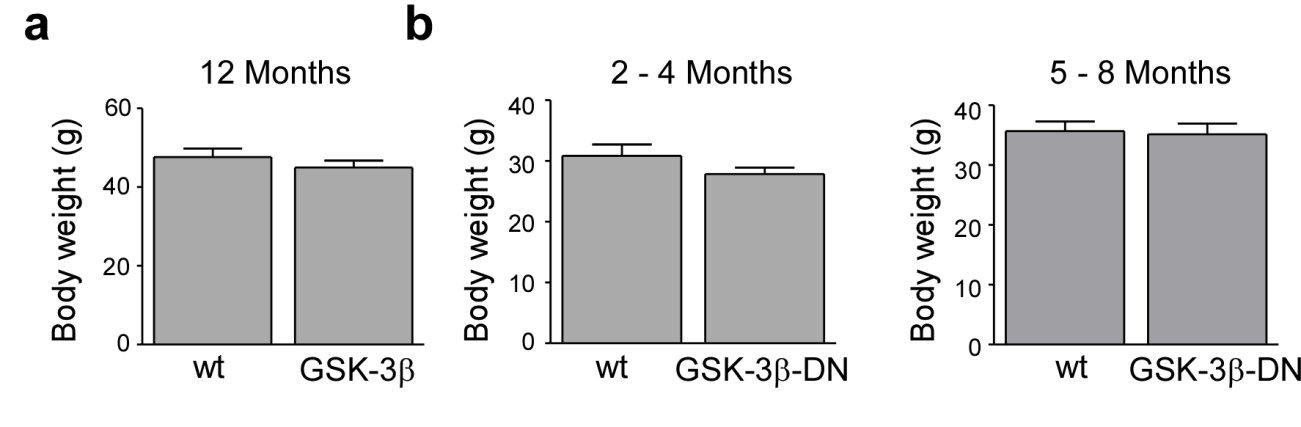


**FigS1:** **Body weight of mice with genetically altered GSK-3 activity. (a)** Graph showing no apparent changes in body weight in male mice overexpressing GSK-3β at 12 month of age when compared to age-matched male wildtype mice (mean ± sd, p = 0.35 by student’s two-tailed *t*-test, n = 8 per group). **(b)** No changes in body weight in male mice expressing GSK-3β-DN when compared to age matched male wild-type mice at 2-4 months of age and at 5-8 months of age (2 – 4 months old mice (mean ± sd, p = 0.17 by student’s two-tailed *t*-test, n = 12 wt and 13 GSK-3β-DN for) and for 5-8 old mice (mean ± sd, p = 0.80 by student’s two-tailed *t*-test, n = 7 wt and 8 GSK-3β-DN).

**Supplementary Figure 2**

**
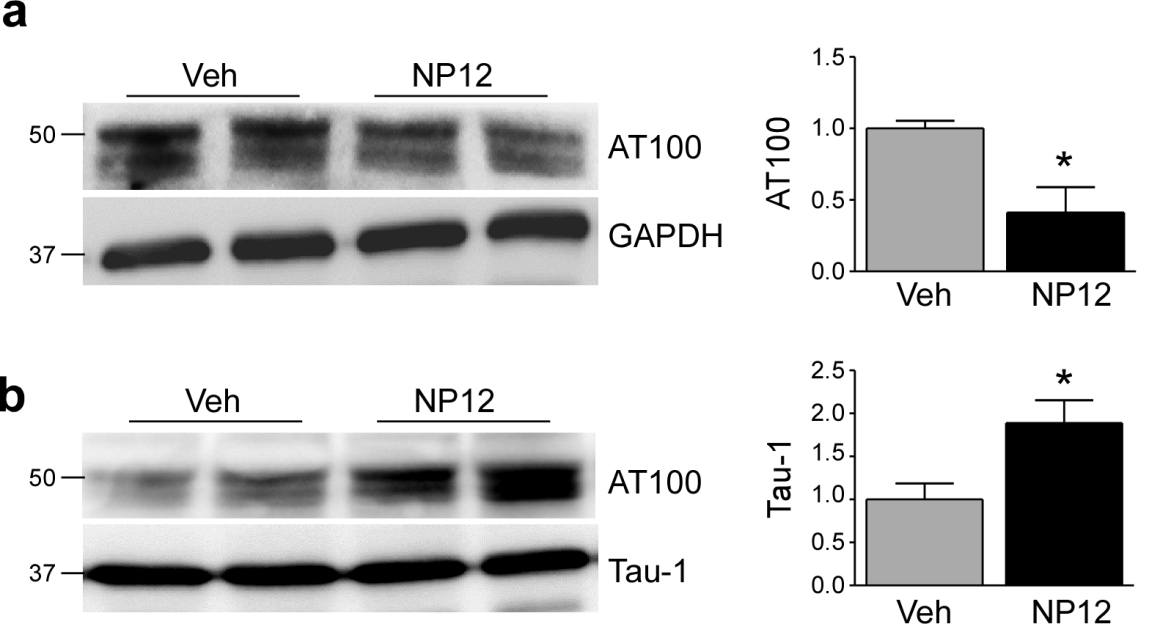
**

# FigS2: Reduced tau phosphorylation following pharmacological GSK-3 inhibition. (a) Representative Western blot (n = 1 per lane) and graphs showing reduced tau phosphorylation detected by the antibody AT100 in hippocampal samples from mice 24 h following treatment with 100 µM NP12 i.c.v.. AT100 detects tau phosphorylated in Ser 212 and Thr 214 (mean ± sd, *p <0.05 by student’s two-tailed *t*-test, n = 4 per group). (b) Western blotting (n = 1 per lane) and graph shows increased reactivity with Tau-1 which detects tau dephosphorization in Ser-199 and -202 in hippocampal samples from mice 24 h following treatment with NP12 100 µM i.c.v. (mean ± sd, *p <0.05 by student’s two-tailed *t*-test, n = 4 per group).
