# Supplementary material for: Bi-directional genetic modulation of GSK-3β exacerbates hippocampal neuropathology in experimental status epilepticus
Source: Cell Death Dis. 2018 Sep 20;9(10):969. doi: 10.1038/s41419-018-0963-5 (PMC6147910; doi:10.1038/s41419-018-0963-5)
Supplement: Supplementary file 2 — Supplementary Information Table 1 [file 41419_2018_963_MOESM2_ESM.docx]

**Supplementary Information:**

**Table 1:** *Genes, implicated in GSK-3 associated pathways with altered expression following status epilepticus*

| **Pathway** | **Up/**  **down** | **Gene name** | **Fold change** |
| --- | --- | --- | --- |
| **PI3-AKT** | **up** | nuclear receptor subfamily 4, group A, member 1 (*Nr4a1*) | 4.58 |
|  |  | oncostatin M receptor (Osmr) | 4.33 |
|  |  | cyclin-dependent kinase inhibitor 1A (P21) (*Cdkn1a*) | 3.95 |
|  |  | interleukin 6 *(Il6*) | 3.76 |
|  |  | myeloid cell leukemia sequence 1 (*Mcl1*) | 3.50 |
|  |  | growth factor receptor bound protein 2 (*Grb2*) | 3.04 |
|  |  | collagen, type IV, alpha 1 (*Col4a1*) | 2.45 |
|  |  | secreted phosphoprotein 1 (*Spp1*) | 2.42 |
|  |  | fibroblast growth factor 3 (*Fgf3*) | 2.12 |
|  |  | myelocytomatosis oncogene (*Myc*) | 1.89 |
|  |  | thrombospondin 1 (*Thbs1*) | 1.91 |
|  |  | protein kinase C, alpha (*Prkca*) | 1.87 |
|  |  | collagen, type V, alpha 2 (*Col5a2*) | 1.86 |
|  |  | hepatocyte growth factor (*Hgf*) | 1.85 |
|  |  | ephrin A1 (*Efna1*) | 1.83 |
|  |  | collagen, type IV, alpha 2 (*Col4a2*) | 1.82 |
|  |  | protein phosphatase 2 (formerly 2A), catalytic subunit, alpha isoform (*Ppp2ca*) | 1.76 |
|  |  | fibroblast growth factor receptor 1 (*Fgfr1*) | 1.74 |
|  |  | collagen, type III, alpha 1 (*Col3a1*) | 1.72 |
|  |  | Janus kinase 1 (*Jak1*) | 1.71 |
|  |  | BCL2-like 11 (apoptosis facilitator) (*Bcl2l11*) | 1.62 |
|  |  | eukaryotic translation initiation factor 4E member 2 (*Eif4e2*) | 1.62 |
|  |  | angiopoietin 2 (*Angpt2*) | 1.60 |
|  |  | cAMP responsive element binding protein 3-like 2 (*Creb3l2*) | 1.58 |
|  |  | collagen, type XI, alpha 1 (*Col11a1*) | 1.58 |
|  |  | guanine nucleotide binding protein (G protein), gamma 2 (*Gng2*) | 1.58 |
|  |  | Kirsten rat sarcoma viral oncogene homolog (*Kras*) | 1.55 |
|  |  | fibroblast growth factor 7 (*Fgf7*) | 1.54 |
|  |  | platelet-derived growth factor, C polypeptide (*Pdgfc*) | 1.54 |
|  |  | toll-like receptor 4 (*Tlr4*) | 1.54 |
|  |  | cAMP responsive element binding protein 1 (*Creb1*) | 1.52 |
|  |  | Ras homolog enriched in brain (*Rheb*) | 1.50 |
|  |  | fibroblast growth factor 10 (*Fgf10*) | 1.50 |
|  |  | platelet derived growth factor, B polypeptide (*Pdgfb*) | 1.50 |
|  | **down** | integrin alpha 4 (*Itga4*) | -1.87 |
|  |  | phosphatidylinositol 3-kinase, regulatory subunit, polypeptide 1 (p85 alpha) (*Pik3r1*) | -1.87 |
|  |  | son of sevenless homolog 2 (Drosophila) (*Sos2*) | -1.84 |
|  |  | tyrosine 3-monooxygenase/tryptophan 5-monooxygenase activation protein, zeta polypeptide (*Ywhaz*) | -1.83 |
|  |  | protein phosphatase 2, regulatory subunit B, gamma (*Ppp2r2c*) | -1.81 |
|  |  | prolactin receptor (*Prlr*) | -1.79 |
|  |  | tyrosine 3-monooxygenase/tryptophan 5-monooxygenase activation protein, epsilon polypeptide (*Ywhae*) | -1.77 |
|  |  | phosphatidylinositol 3-kinase, catalytic, alpha polypeptide (*Pik3ca*) | -1.62 |
|  |  | lysophosphatidic acid receptor 4 (*Lpar4*) | -1.61 |
|  |  | integrin alpha 8 (*Itga8*) | -1.56 |
|  |  | erythropoietin receptor (*Epor*) | -1.53 |
|  |  | growth hormone (*Gh*) | -1.52 |
|  |  | fibroblast growth factor receptor 3 (*Fgfr3*) | -1.51 |
|  |  | activating transcription factor 2 (*Atf2*) | -1.50 |
| **Insulin** | **up** | suppressor of cytokine signaling 3 (*Socs3*) | 6.70 |
|  |  | growth factor receptor bound protein 2 (*Grb2*) | 3.04 |
|  |  | SHC (Src homology 2 domain containing) family, member 4 (*Shc4*) | 2.01 |
|  |  | thyroid hormone receptor interactor 10 (*Trip10*) | 1.88 |
|  |  | hexokinase 2 (*Hk2*) | 1.84 |
|  |  | insulin receptor substrate 2 (*Irs2*) | 1.82 |
|  |  | protein kinase, AMP-activated, beta 2 non-catalytic subunit (*Prkab2*) | 1.82 |
|  |  | MAP kinase-interacting serine/threonine kinase 2 (*Mknk2*) | 1.75 |
|  |  | protein kinase, cAMP dependent regulatory, type II alpha (*Prkar2a*) | 1.65 |
|  |  | suppressor of cytokine signaling 1 (*Socs1*) | 1.64 |
|  |  | protein phosphatase 1, regulatory (inhibitor) subunit 3C (*Ppp1r3c*) | 1.63 |
|  |  | eukaryotic translation initiation factor 4E member 2 (*Eif4e2*) | 1.62 |
|  |  | Braf transforming gene (*Braf*) | 1.62 |
|  |  | Kirsten rat sarcoma viral oncogene homolog (*Kras*) | 1.56 |
|  |  | protein tyrosine phosphatase, non-receptor type 1 (*Ptpn1*) | 1.53 |
|  |  | protein kinase, cAMP dependent regulatory, type I, alpha (*Prkar1a*) | 1.53 |
|  |  | Ras homolog enriched in brain (*Rheb*) | 1.50 |
|  | **down** | Araf proto-oncogene, serine/threonine kinase (*Araf*) | -2.76 |
|  |  | phosphatidylinositol 3-kinase, regulatory subunit, polypeptide 1 (p85 alpha) (*Pik3r1*) | -1.87 |
|  |  | son of sevenless homolog 2 (Drosophila) (*Sos2*) | -1.84 |
|  |  | protein phosphatase 1, catalytic subunit, beta isoform (*Ppp1cb*) | -1.75 |
|  |  | phosphatidylinositol 3-kinase, catalytic, alpha polypeptide (*Pik3ca*) | -1.62 |
|  |  | protein kinase, cAMP dependent regulatory, type II beta (*Prkar2b*) | -1.62 |
| **WNT** | **up** | fos-like antigen 1 (*Fosl1*) | 3.06 |
|  |  | wingless-type MMTV integration site family, member 9B (*Wnt9b*) | 2.09 |
|  |  | myelocytomatosis oncogene (*Myc*) | 1.89 |
|  |  | prickle planar cell polarity protein 1 (*Prickle1*) | 1.88 |
|  |  | protein kinase C, alpha (*Prkca*) | 1.87 |
|  |  | frizzled class receptor 3 (*Fzd3*) | 1.81 |
|  |  | jun proto-oncogene (*Jun*) | 1.81 |
|  |  | SUMO/sentrin specific peptidase 2 (*Senp2*) | 1.6 |
|  |  | ras homolog family member A (*Rhoa*) | 1.5 |
|  | **down** | calcium/calmodulin-dependent protein kinase II, delta (*Camk2d*) | -2.39 |
|  |  | secreted frizzled-related protein 1 (*Sfrp1*) | -1.71 |
|  |  | phospholipase C, beta 4 (*Plcb4*) | -1.66 |
|  |  | wingless-type MMTV integration site family, member 9A (*Wnt9a*) | -1.57 |
|  |  | calcium/calmodulin-dependent protein kinase II gamma (*Camk2g*) | -1.54 |
|  |  | vang-like 2 (van gogh, Drosophila) (*Vangl2*) | -1.50 |
| **mTor** | **up** | ribosomal protein S6 kinase polypeptide 3 (*Rps6ka3*) | 2.19 |
|  |  | protein kinase C, alpha (*Prkca*) | 1.87 |
|  |  | eukaryotic translation initiation factor 4E member 2 (*Eif4e2*) | 1.62 |
|  |  | Braf transforming gene (*Braf*) | 1.61 |
|  |  | Ras homolog enriched in brain (*Rheb*) | 1.51 |
|  | **down** | phosphatidylinositol 3-kinase, regulatory subunit, polypeptide 1 (p85 alpha) (*Pik3r1*) | -1.87 |
|  |  | phosphatidylinositol 3-kinase, catalytic, alpha polypeptide (*Pik3ca*) | -1.62 |
