# Supplementary material for: Bi-directional genetic modulation of GSK-3β exacerbates hippocampal neuropathology in experimental status epilepticus
Source: Cell Death Dis. 2018 Sep 20;9(10):969. doi: 10.1038/s41419-018-0963-5 (PMC6147910; doi:10.1038/s41419-018-0963-5)
Supplement: Supplementary file 3 — Supplementary Information Table 2 [file 41419_2018_963_MOESM3_ESM.docx]

**Supplementary Information:**

**Table 2:** *Altered pathways and associated genes in mice overexpressing GSK-3β following status epilepticus*

(i) Top ten biological functions with associated up-regulated genes in GSK-3β overexpressing mice post-status epilepticus according to combined score ranking using Enrichr:

| **Biological Function** | **Gene symbol** |
| --- | --- |
| neutrophil degranulation | ITGAM;TNFAIP6;RAB3D;SNAP23;MOSPD2;CXCL1;FPR2;ORM2;CXCL5;C3;PLAC8;PYCARD;GLIPR1;CYB5R3;GM2A;CLEC5A;CTSH;SLC15A4;CAP1;CD93;CYBB;CYBA;MMP8;BST2;IL6;RAB31;CLEC4D;PTPRC;TYROBP;LCN2;RAB18;CHI3L1;PTX3;S100A9;HSPA1B;S100A8;HSPA1A;TLR2 |
| cellular response to interferon-gamma | GBP6;CCL25;IFITM3;IFITM1;IFITM2;GCH1;CCL20;CXCL16;BST2;CCL7;CCL5;HCLS1;CCL2;CCL19;LGALS9;IL12RB1;TRIM21 |
| chronic inflammatory response | CCL25;TNFAIP6;CCL20;TNFRSF18;CYBB;CYBA;CXCL1;FPR2;CXCL13;CXCL5;CXCL10;CXCL11;IL6;CCL7;CCL5;CRH;CHI3L1;CCL2;PTX3;LGALS9;CCL19;S100A9;S100A8 |
| inflammatory response to wounding | CCL25;TNFAIP6;CCL20;TNFRSF18;CYBB;CYBA;CXCL1;FPR2;CXCL13;CXCL5;CXCL10;CXCL11;IL6;CCL7;CCL5;CRH;CHI3L1;CCL2;PTX3;LGALS9;CCL19;S100A9;S100A8 |
| inflammatory response | CCL25;TNFAIP6;CCL20;TNFRSF18;CYBB;CYBA;CXCL1;FPR2;CXCL13;CXCL5;CXCL10;CXCL11;IL6;CCL7;CCL5;CRH;CHI3L1;CCL2;PTX3;LGALS9;CCL19;S100A9;S100A8 |
| acute inflammatory response | CCL25;TNFAIP6;CCL20;TNFRSF18;CYBB;CYBA;CXCL1;FPR2;CXCL13;CXCL5;CXCL10;CXCL11;IL6;CCL7;CCL5;CRH;CHI3L1;CCL2;PTX3;LGALS9;CCL19;S100A9;S100A8 |
| type I interferon signaling pathway | IFITM3;IFITM1;IFITM2;RSAD2;STAT1;MX2;MX1;IFI35;IFIT1;IFIT3;PSMB8;IFIT2;ISG20;BST2;IL6;CCL2;IL6ST;GBP2;IL12RB1;XAF1 |
| negative regulation of cell proliferation | RARG;IFITM1;SLA;CXCL1;BRCA1;CXCL13;ETS1;IFIT3;HNF4A;MXI1;CDKN2B;TNFRSF18;EIF2AK2;PPP2R5C;OPRM1;TGFBR2;CXCL10;CXCL11;SFRP1;CNOT7;CDK6;FABP7;BTK;SCGB3A1;SGK3;HSPA1B;HSPA1A;CDKN3 |
| positive regulation of ERK1 and ERK2 cascade | CCL25;FLT1;CCL20;PELI2;LPAR1;OPRM1;FGF1;CYR61;ICAM1;PYCARD;IL6;CCL7;GLIPR2;GPR183;CCL5;CHI3L1;CCL2;LGALS9;CCL19;SLAMF1 |
| chemokine (C-C motif) ligand 5 signaling pathway | CCL25;CXCL10;CXCL11;CCL7;CCL20;GPR35;CCL5;CCL2;CXCL1;CCL19;CXCL13;CXCL5 |

(ii) Top ten biological functions associated with down-regulated genes in GSK-3β overexpressing mice post-status epilepticus according to combined score ranking using Enrichr:

| **Biological function** | **Gene symbol** |
| --- | --- |
| neuron-neuron synaptic transmission | CHRNA5;NRXN1;KCNC4;PTEN;GRIK4;GRM2;GRM5;GRM4;NPY;PENK;KIF1B;DLGAP1;NPTX1;NPTX2;UNC13B;HOMER1;KCND2;SYT1;NPY1R;PDYN;GRIN2B;SSTR3;AKAP9;KCNQ2;KCNQ5;CBLN1 |
| chemical synaptic transmission, postsynaptic | GSK3B;CHRNA5;NRXN1;KCNC4;GRIK4;GRM2;GRM5;GRM4;NPY;PENK;DLGAP1;NPTX1;NPTX2;UNC13B;HOMER1;KCND2;SYT1;NPY1Y;PDYN;GRIN2B;SSTR3;AKAP9;KCNQ2;KCNQ5;CBLN1 |
| synaptic transmission, dopaminergic | CHRNA5;NRXN1;KCNC4;GRIK4;CRHBP;GRM2;GRM5;GRM4;NPY;PENK;DLGAP1;NPTX1;NPTX2;UNC12B;HOMER1;KCND2;SYT1;NPY1R;PDYN;GRIN2B;SSTR3;AKAP9;KCNQ2;KCNQ5;CBLN1 |
| neuromuscular synaptic transmission | CHRNA5;NRXN1;KCNC4;GRIK4;GRM2;GRM5;GRM4;NPY;PENK;KIF1B;DLGAP1;NPTX1;NPTX2;UNC13B;HOMER1;KCND2;SYT1;NPY1R;PDYN;GRIN2B;SSTR3;AKAP9;KCNQ2;KCNQ5;CBLN1 |
| synaptic transmission, noradrenergic | UNC13B;HOMER1;CHRNA5;KCND2;SYT1;NRXN1;KCNC4;NPY1R;GRIK4;PDYN;GRIN2B;SSTR3;GRM2;GRM5;GRM4;NPY;AKAP9;PENK;KCNQ2;DLGAP1;KCNQ5;NPTX1;NPTX2;CBLN1 |
| synaptic transmission, serotonergic | UNC13B;HOMER1;CHRNA5;KCND2;SYT1;NRXN1;KCNC4;NPY1R;GRIK4;PDYN;GRIN2B;SSTR3;GRM2;GRM5;GRM4;NPY;AKAP9;PENK;KCNQ2;DLGAP1;KCNQ5;NPTX1;NPTX2;CBLN1 |
| chemical synaptic transmission | UNC13B;HOMER1;CHRNA5;KCND2;SYT1;NRXN1;KCNC4;NPY1R;GRIK4;PDYN;GRIN2B;SSTR3;GRM2;GRM5;GRM4;NPY;AKAP9;PENK;KCNQ2;DLGAP1;KCNQ5;NPTX1;NPTX2;CBLN1 |
| excitatory chemical synaptic transmission | UNC13B;HOMER1;CHRNA5;KCND2;SYT1;NRXN1;KCNC4;NPY1R;GRIK4;PDYN;GRIN2B;SSTR3;GRM2;GRM5;GRM4;NPY;AKAP9;PENK;KCNQ2;DLGAP1;KCNQ5;NPTX1;NPTX2;CBLN1 |
| phenylethylamine metabolic process involved in synaptic transmission | UNC13B;HOMER1;CHRNA5;KCND2;SYT1;NRXN1;KCNC4;NPY1R;GRIK4;PDYN;GRIN2B;SSTR3;GRM2;GRM5;GRM4;NPY;AKAP9;PENK;KCNQ2;DLGAP1;KCNQ5;NPTX1;NPTX2;CBLN1 |
| spontaneous synaptic transmission | UNC13B;HOMER1;CHRNA5;KCND2;SYT1;NRXN1;KCNC4;NPY1R;GRIK4;PDYN;GRIN2B;SSTR3;GRM2;GRM5;GRM4;NPY;AKAP9;PENK;KCNQ2;DLGAP1;KCNQ5;NPTX1;NPTX2;CBLN1 |
